# Themed issue: Inflammation, repair and ageing

**DOI:** 10.1111/bph.15799

**Published:** 2022-02-17

**Authors:** Claudio Mauro, Amy J. Naylor, Janet M. Lord

**Affiliations:** ^1^ Institute of Inflammation and Ageing University of Birmingham Birmingham UK

## LINKED ARTICLES

This article is part of a themed issue on Inflammation, Repair and Ageing. To view the other articles in this section visit http://onlinelibrary.wiley.com/doi/10.1111/bph.v179.9/issuetoc

In this themed issue, we have assembled contributions from researchers studying various aspects of inflammation and tissue repair as they relate to ageing. Ageing can be defined in biological terms as *the increased frailty of an organism with time that reduces resilience to stress, increasing the chance of disease and death*. Importantly, the processes driving the aged phenotype are now becoming understood, with nine hallmarks of ageing proposed (Lopez‐Otin et al., 2013). The hallmarks are hierarchical, initiated by cellular damage and culminating in increased inflammation and a reduced ability to regenerate tissues through stem cell activity. This issue considers some of the drivers of these end‐stage processes in ageing, including the aged, proinflammatory immune system.

Advancing age is accompanied by significant remodelling of the immune system (immunesenescence), increased systemic inflammation (inflammageing) and metabolic alterations including insulin resistance, hypertension and dyslipidaemia. All of these contribute to an increased risk of chronic inflammatory diseases as well as reduced capacity for repair, in older adults. The lungs are highly susceptible to cell senescence—of lung tissue cells as well as resident immune cells. Senescent cells have a proinflammatory secretome that contributes to a proinflammatory environment and a reduced capacity to deal with infectious challenges. Consequently, lung diseases disproportionately affect older individuals, with 70% of hospital‐treated community‐acquired pneumonia occurring in those aged over 65. In their review, ‘Inflammation, ageing and diseases of the lung: Potential therapeutic strategies from shared biological pathways’, Faniyi et al. (2022) make the case that understanding the common processes that underlie seemingly disparate and unrelated lung pathologies may provide a common route to treatment. Indeed, the ongoing COVID‐19 pandemic serves as a timely reminder of ageing as a major risk factor for infection. It has brought a heightened understanding of the burden of lung disease and of inflammageing in older adults and an increased interest in drugs which target ageing processes across disease. The unique environment of the lung and the structural and functional changes that occur with advancing age provide challenges, but also opportunities, for treatment by targeting ageing hallmarks.

In their article, ‘Understanding the role of host metabolites in the induction of immunesenescence: Future strategies for keeping the ageing population healthy’, Conway et al. (2022) focus on the potential of metabolic immunotherapeutic strategies to counter immunesenescence and restore immune homeostasis. The article covers host metabolites as inducers of immunesenescence, in particular, saturated fatty acids and cholesterol, ceramides, lactate and the role of amino acids (e.g., glutamine, arginine and tryptophan) as signalling molecules. Increasing evidence of the role of these metabolites in immunomodulation leads inevitably to strategies that attempt to restore immune homeostasis by modulating their availability. Some such interventions are already considered mainstream, such as increased intake of omega‐3 polyunsaturated fatty acids, caloric restriction and routine use of statins to control serum cholesterol levels. Li et al. (2022) tested whether the naturally occurring flavonoid ‘sodium rutin improves hepatic fitness and extends life and health span in mice’. They demonstrated that treatment extended the lifespan of mice by 10% and that whole‐body metabolism was affected, as demonstrated by increased energy expenditure, lower respiratory quotient and enhanced autophagy in hepatocytes. Navarro et al. (2022) delved into another aspect of metabolism that contributes to inflammatory conditions and inflammageing in their review article, ‘nicotinamide adenine dinucleotide (NAD) metabolism in the immune response, autoimmunity and inflammageing’. NAD+ is an enzyme cofactor in redox reactions and therefore a critical metabolic intermediate; however, NAD+ levels decline in several tissues with age. Its role as a co‐substrate for enzymes such as the sirtuins, adenosine diphosphate ribose transferases and synthases establishes it as a regulator of multiple cellular functions including energy metabolism, DNA repair, regulation of the epigenetic landscape and inflammation, all of which have the potential to impact on healthy ageing and repair processes.

The central role of macrophages as orchestrators of inflammation and repair across almost all tissues makes them particularly promising targets for therapeutic intervention. Raucci et al. (2022) share their novel findings that ‘IL‐17 induced inflammation modulates mPGES‐1/PPAR‐γ pathway in monocytes/macrophages’, leading them to suggest that IL‐17 constitutes a specific modulator of inflammatory monocytes during later phases of the inflammatory response and that the IL‐17/mPGES‐1/PPAR‐γ pathway represents a potential therapeutic target for inflammatory and immune‐mediated diseases. In their original research articles, Olona, Hateley, Guerrero, et al. (2022) describe their findings that ‘cardiac glycosides cause selective cytotoxicity in human macrophages and ameliorate white adipose tissue homeostasis’ and Xing et al. (2022) demonstrate that ‘convallatoxin inhibits IL‐1β production by suppressing zinc finger protein 91‐mediated pro‐IL‐1β ubiquitination and caspase‐8 inflammasome activity’. These studies reveal novel anti‐inflammatory drug targets that have potential as inhibitors of the inappropriate immune response seen during inflammation and ageing.

Two of the articles included in this collection have the impact of ageing on vision as their focus. In their review, ‘emerging therapies and their delivery for treating age‐related macular degeneration’, Thomas et al. (2022) describe age‐related macular degeneration (AMD), the most common cause of blindness in the Western world. With new therapeutic strategies emerging for AMD, novel delivery routes and their implications for translation into clinical practice are critical factors in determining their future success. One such promising agent is highlighted in the original research paper by Ju et al. (2022), entitled ‘Protection against light‐induced retinal degeneration via dual anti‐inflammatory and anti‐angiogenic functions of thrombospondin‐1’. Their finding that Thrombospondin‐1 was protective against blue light‐induced retinal inflammation and angiogenesis by blocking the activated NF‐κB and VEGFR2 pathways, respectively, is promising.

In each of these articles, the key role of immunesenescence and altered metabolism in contributing to inflammageing, and how they in turn compromise repair processes, is evident. Indeed, studies in mice have revealed that inducing senescence in T cells through compromised mitochondrial function is sufficient to lead to multimorbidity and frailty (Desdín‐Mico et al., 2020). An aged immune system may thus be a prime driver of the aged phenotype, suggesting that targeting this process may provide a novel route to preventing many age‐related diseases and frailty.

## CONFLICT OF INTEREST

The authors wish to acknowledge that CM and JL have co‐authored a paper included within this special issue (Conway et al.). In addition, CM has co‐authored papers with J. Behmoaras.

## References

[bph15799-bib-0001] Conway, J. , Certo, M. , Lord, J. M. , Mauro, C. , & Duggal, N. A. (2022). Understanding the role of host metabolites in the induction of immunesenescence: Future strategies for keeping the ageing population healthy. British Journal of Pharmacology, 179(9), 1808–1824. 10.1111/bph.15671 34435354

[bph15799-bib-0002] Desdín‐Mico, G. , Soto‐Heredero, G. , Aranda, J. F. , Oller, J. , Carrasco, E. , Gabandé‐Rodríguez, E. , Blanco, E. M. , Alfranca, A. , Cuss_o, L. , Desco, M. , & Ibañez, B. (2020). T cells with dysfunctional mitochondria induce multimorbidity and premature senescence. Science, 368, 1371–1376. 10.1126/science.aax0860 32439659 PMC7616968

[bph15799-bib-0003] Faniyi, A. A. , Hughes, M. J. , Scott, A. , Belchamber, K. B. R. , & Sapey, E. (2022). Inflammation, ageing and diseases of the lung: Potential therapeutic strategies from shared biological pathways. British Journal of Pharmacology, 179(9), 1790–1807. 10.1111/bph.15759 34826882

[bph15799-bib-0004] Ju, Y. , Tang, Z. , Dai, X. , Gao, H. , Zhang, J. , Liu, Y. , Yang, Y. , Ni, N. , Zhang, D. , Wang, Y. , Sun, N. , Yin, L. , Luo, M. , Zhang, J. , & Gu, P. (2022). Protection against light‐induced retinal degeneration via dual anti‐inflammatory and anti‐angiogenic functions of thrombospondin‐1. British Journal of Pharmacology, 179(9), 1938–1961. 10.1111/bph.15303 33125704

[bph15799-bib-0005] Li, S. , Li, J. , Pan, R. , Cheng, J. , Cui, Q. , Chen, J. , & Yuan, Z. (2022). Sodium rutin extends lifespan and health span in mice including positive impacts on liver health. British Journal of Pharmacology, 179(9), 1825–1838. 10.1111/bph.15410 33555034

[bph15799-bib-0006] Lopez‐Otin, C. , Blasco, M. A. , Partridge, L. , Serrano, M. , & Kroemer, G. (2013). The hallmarks of aging. Cell, 153, 1194–1217. 10.1016/j.cell.2013.05.039 23746838 PMC3836174

[bph15799-bib-0007] Navarro, M. N. , Gomez de las Heras, M. M. , & Mittelbrunn, M. (2022). Nicotinamide adenine dinucleotide metabolism in the immune response, autoimmunity and inflammageing. British Journal of Pharmacology, 179(9), 1839–1856. 10.1111/bph.15477 33817782 PMC9292562

[bph15799-bib-0008] Olona, A. , Hateley, C. , Guerrero, A. , Ko, J.‐H. , Johnson, M. R. , Anand, P. K. , Thomas, T. , Gil, J. , & Behmoaras, J. (2022). Cardiac glycosides cause cytotoxicity in human macrophages and ameliorate white adipose tissue homeostasis. British Journal of Pharmacology, 179(9), 1874–1886. 10.1111/bph.15423 33665823

[bph15799-bib-0010] Raucci, F. , Saviano, A. , Casillo, G. M. , Guerra‐Rodriguez, M. , Mansour, A. A. , Piccolo, M. , Ferraro, M. G. , Panza, E. , Vellecco, V. , Irace, C. , Caso, F. , Scarpa, R. , Mascolo, N. , Alfaifi, M. , Iqbal, A. J. , & Maione, F. (2022). IL‐17‐induced inflammation modulates the mPGES‐1/PPAR‐γ pathway in monocytes/macrophages. British Journal of Pharmacology, 179(9), 1857–1873. 10.1111/bph.15413 33595097

[bph15799-bib-0011] Thomas, C. N. , Sim, D. A. , Lee, W. H. , Alfahad, N. , Dick, A. D. , Denniston, A. K. , & Hill, L. J. (2022). Emerging therapies and their delivery for treating age‐related macular degeneration. British Journal of Pharmacology, 179(9), 1908–1937. 10.1111/bph.15459 33769566

[bph15799-bib-0012] Xing, Y. , Wang, J. Y. , Li, M. Y. , Zhang, Z. H. , Jin, H. L. , Zuo, H. X. , Ma, J. , & Jin, X. (2022). Convallatoxin inhibits IL‐1β production by suppressing zinc finger protein 91‐mediated pro‐IL‐1β ubiquitination and caspase‐8 inflammasome activity. British Journal of Pharmacology, 179(9), 1887–1907. 10.1111/bph.15758 34825365

